# Experimental Study on Rebar Corrosion Using the Galvanic Sensor Combined with the Electronic Resistance Technique

**DOI:** 10.3390/s16091451

**Published:** 2016-09-08

**Authors:** Yunze Xu, Kaiqiang Li, Liang Liu, Lujia Yang, Xiaona Wang, Yi Huang

**Affiliations:** 1 School of Naval Architecture and Ocean Engineering, Dalian University of Technology, Dalian 116024, China; liuliang93@126.com (L.L.); ylj-yang@163.com (L.Y.); 2 School of Physics and Optoelectronic Engineering, Dalian University of Technology, Dalian 116024, China; likaiqiang_dlut@163.com

**Keywords:** rebar corrosion, galvanic sensor, localized corrosion, ER, LPR

## Abstract

In this paper, a new kind of carbon steel (CS) and stainless steel (SS) galvanic sensor system was developed for the study of rebar corrosion in different pore solution conditions. Through the special design of the CS and SS electronic coupons, the electronic resistance (ER) method and zero resistance ammeter (ZRA) technique were used simultaneously for the measurement of both the galvanic current and the corrosion depth. The corrosion processes in different solution conditions were also studied by linear polarization resistance (LPR) and the measurements of polarization curves. The test result shows that the galvanic current noise can provide detailed information of the corrosion processes. When localized corrosion occurs, the corrosion rate measured by the ER method is lower than the real corrosion rate. However, the value measured by the LPR method is higher than the real corrosion rate. The galvanic current and the corrosion current measured by the LPR method shows linear correlation in chloride-containing saturated Ca(OH)_2_ solution. The relationship between the corrosion current differences measured by the CS electronic coupons and the galvanic current between the CS and SS electronic coupons can also be used to evaluate the localized corrosion in reinforced concrete.

## 1. Introduction

Reinforced concrete is one of the most useful construction materials around the world due to its durable and strong performance throughout its service life [1]. The carbon steel (CS) rebar buried in concrete is passive by forming a thin layer of hydroxides in the alkaline conditions [2]. However, due to acid rain, sea water immersion, and overuse of de-icing salts, the presence of aggressive ions in concrete structures, such as chloride and sulfate ions, may lead to the breakdown of the passive film on the steel rebar [3,4,5]. In addition, the carbon dioxide in the air also can react with the alkaline compounds in the pore solution and result in the decrease of pH, which may also cause the damage to the passive film [3,6]. Once local breakdown of passive film on the steel surface occurs, the steel without passive film will suffer a much lower potential than the other parts of the rebar where passive film still exists [7]. Consequently, the area without passive film will act as anode, and serious pitting corrosion may happen on the rebar. To promote the reliability of the reinforced concrete, stainless steel (SS, such as 316L and 304L steels, have been used to minimize reinforcement corrosion in some conditions [8]. However, due to the high cost of the SS, it can only be used to replace CS in the part of the rebar subject to a harsh environment. Therefore, galvanic corrosion between SS and CS becomes another question when the two kinds of steels are used simultaneously in a concrete structure [9,10].

To monitor the corrosion process of the rebar in mortars, various embedded sensors have been developed [11,12,13]. Since rebar corrosion always presents as an electrochemical process, traditional electrochemical techniques such as half-cell potential, linear polarization resistance (LPR), and electrochemical impedance spectrum (EIS) measurements are widely applied in concrete structures [14]. The half-cell potential measurements can be performed with embedded or external reference electrodes without polarizing the steel electrode. Due to its simplicity of operation, measurements of half-cell potentials are most frequently used in the field [12]. However, potential measurement results cannot give any information about the actual kinetics of the corrosion process. When this method is used in an underwater zone, the half-cell potential measurement results may misinterpret the corrosion status due to the low oxygen concentration [5,13]. Though the polarization resistance, double-layer capacitance and ohmic resistivity of the concrete can be measured by LPR and EIS methods, the high resistance of the concrete gives a large limitation to these methods being used in practical engineering [15]. In recent studies, electronic resistance (ER) probes, which are popular for the pipeline industry [16], have been applied in concrete structures. The metal loss of the rebar can be directly calculated from the resistance change of the probe, and an electrolyte layer on the metal surface is not inevitable during ER measurements [17]. Therefore, ER probes provide more definite results compared with traditional electrochemical methods in mortars. The limitation of the ER sensor is that it can only provide accurate metal loss of uniform corrosion patterns. Narrow but deep pitting corrosion can occur without any significant metal loss and resistance change of the sensor [18]. Therefore, ER sensors will be unreliable once localized corrosion happens. In order to monitor the propagation of the localized corrosion in concrete structures, Legat [7,19] adopted both ER probes and the electrochemical noise (EN) method for the average corrosion rate measurement and the monitoring of the variation of current and potential noises. From Legat’s test, the degradation of the passive film and the initial metastable pitting corrosion were well-detected by the transients of the current and potential changes. Though the EN signal has a good correlation with pitting corrosion or crevice corrosion, the EN signal is vulnerable to interference from other electronic noise and the EN measurement results are still controversial in the explanation of the localized corrosion processes [7,20]. Another localized corrosion monitoring sensor, which is called the wire beam electrode (WBE), is also applied in buried conditions [21,22]. The WBE can simulate the corrosion processes on a simulated one-piece electrode by continuously mapping the potential and galvanic current on the surface of the WBE [23]. Dong [24] has used WBE to study the influence of the rust layer for rebar corrosion in a concrete block. The measurement results showed that the expansion of the rust layer can initiate new pitting corrosion on the surface of passive wire electrodes. The WBE can provide more accurate localized corrosion information compared to the EN method, but the WBE is much more complex in fabrication due to its 100 tiny electrodes and it cannot directly reflect the corrosion rates of the rebar in mortar [25].

The galvanic corrosion sensor has received more attention in recent years due to its high sensitivity and easy access for operators [26,27]. Galvanic sensors which also can be called galvanic macrocell sensors always consist of two dissimilar metals [28]. The galvanic current between the two different kinds of metals is sensitive to the changes of the corrosion conditions, such as oxygen concentration, temperature, the passivation state of the metal, and so on [29]. Through the measurement of the galvanic current, the initiation and variation of the corrosion in concrete can be well-reflected. Some studies even found that the galvanic current has a strong correlation to the corrosion rate of the rebar using pore solution [28,30]. Yoo [30] has used two different kinds of galvanic sensors (steel/stainless galvanic sensor and steel/copper galvanic sensor) in pore solutions. The output current of both galvanic sensors showed linear correlations compared to the weight loss measurement result. In his further studies [28], the steel/copper galvanic sensor still kept a good linear response to the corrosion rate measured by the LPR method in mortar. Pereira [31] found that the logarithmic values of the galvanic current provided by the steel/stainless sensor and the corrosion current also had a linear correlation in carbonate pore solution conditions with different solution temperatures. The test results illustrated that galvanic sensors can be used for corrosion rates monitoring in concrete structures. Meanwhile, the galvanic corrosion behavior between the CS and SS can be monitored by the steel/stainless sensor. The galvanic current variation is also an excellent indicator for the breakdown of the passive film and the initiation of localized corrosion [32]. More detailed information of the corrosion processes can be expressed by galvanic sensors. It is also quite easy for fabrication compared to the conventional sensors mentioned above. However, some researchers consider that the macrocell current between the two dissimilar metals cannot directly reflect the corrosion current due the existence of the microcell current on the steel electrode itself [26]. Thus, more experimental tests and improvements should be carried out on galvanic sensors for the monitoring of rebar corrosion in mortars.

In this paper, a new design steel/stainless (CS/SS) galvanic sensor was developed for the monitoring of rebar corrosion by using three electronic coupons. Besides the galvanic current monitoring, the corrosion depths of the three coupons can be monitored by the four wires ER method. Based on this new technique, more information can be obtained through the whole corrosion process. The new sensor was tested in simulated pore solution with different chloride ion concentrations and pH. The galvanic current and the corrosion depths measured by the new sensor were compared with the corrosion current measured by the LPR method. The galvanic corrosion behaviors between CS and SS in different conditions were also studied by the new design galvanic sensor.

## 2. Experimental Methods

### 2.1. Materials of the Electronic Coupons and the Electrodes

**Figure 1:**
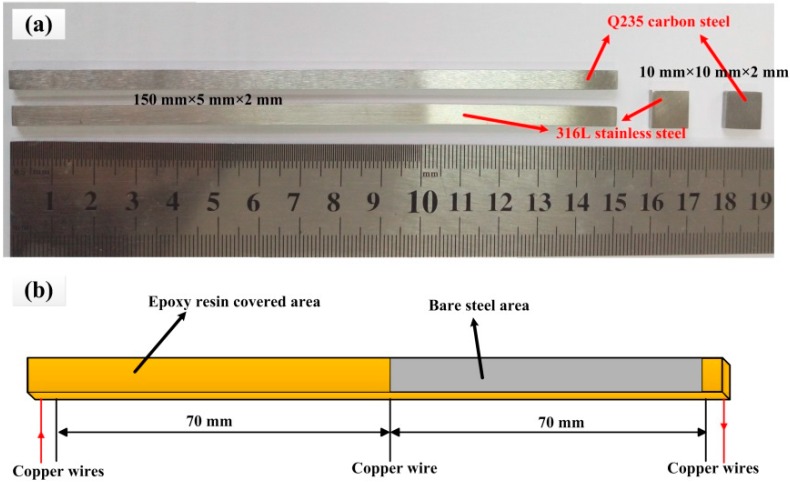
Electronic coupons and electrodes used in tests (**a**) the photo of electronic coupons and electrodes (**b**) the structure of the electronic coupon.

The materials used in the test were Q235 carbon steel and 316L stainless steel. The chemical compositions of the steels are shown in Table 1. The specimens with different dimensions used in the tests are shown in Figure 1a. The specimens machined in the dimensions of 150 mm × 5 mm × 2 mm were used as electronic coupons. The detailed structure of the electronic coupon is shown in Figure 1b. It is seen that the electronic coupon was divided into two parts with the same lengths. A half part of the electronic coupon was completely sealed with epoxy resin and the other half part had a 70 mm long bare steel area on the front face. Five copper wires were welded at the back side of the electronic coupon. The two copper wires on the edges of the electronic coupon were used for current injecting and the other three coppers were used for voltage measurements. The small specimens used for electrochemical tests were made in a dimension of 10 mm × 10 mm × 2 mm. The specimens were sealed with epoxy resin with the end face exposed to the solution. A copper wire was welded at the back side of the specimen and it was also sealed in the epoxy. Before the test, the working surface of the electronic coupons and electrodes were ground consequentially from 600 to 1200 grit silicon carbide paper, rinsed with deionized water and degreased in acetone.

### 2.2. The Measurement Principle of the Galvanic Sensor System

**Figure 2:**
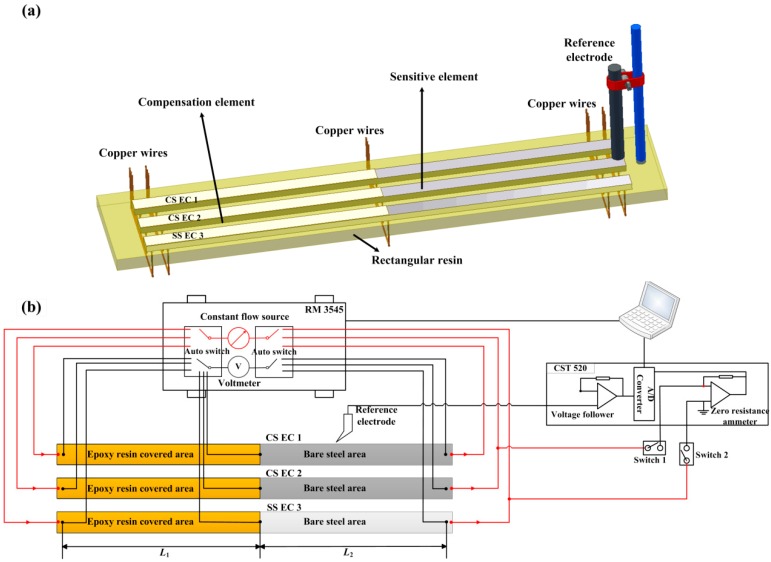
The galvanic sensor system (**a**) scheme of the sensor (**b**) measurement circuit of the sensor system.

It is seen from Figure 2a that the sensor system consists of three electronic coupons. Two of the coupons were carbon steel electronic coupons (CS EC 1 and CS EC 2) and one of the coupons was a stainless steel electronic coupon (SS EC 3). The electronic coupons were embedded in a rectangular resin with their measurement wires passing through the back of the rectangular resin. Excepting half parts of the front faces of the electronic coupons, which were kept bare, the other sections of the electronic coupons were all sealed by epoxy resin. A reference electrode was fixed on the bare steel sides of the coupons. The measurement circuit of the galvanic sensor system is shown in Figure 2b. It can be seen that the galvanic sensor system was composed of two monitoring parts, i.e., the corrosion depths monitoring of the electronic coupons by using the RM 3545 Micro Resistance measurement device (HIOKI, Ueda, Japan) and the galvanic current and coupled potential monitoring by using the zero resistance ammeter (ZRA) and high resistance voltage follower (CST 520, CorroTest, Wuhan, China).

The measurement principle of the corrosion depth for each electronic coupon is the same as the thin film ER sensors and the ER probes [18]. The half part of the coupon sealed by epoxy was used as a temperature compensation element and the other bare half part was used as a sensitive element. Because of the measurement error and the size of the weld point, the lengths of the two parts on the electronic coupon may have a tiny difference. Supposing that the length of the compensation element was *L_1_*, the electronic resistance *R_c_* of this element can be expressed as:

(1) $$R_{c}=\rho L_{1}/wd_{0}$$

where *ρ* is the electrical resistivity of the carbon steel or stainless steel, *w* is the width of the coupon and *d*_0_ is the initial thickness of the coupon. Because the compensation element is sealed by epoxy resin, no corrosion happens on this element. The thickness of this part will not change in the whole test. The same as the compensation element, the electronic resistance *R_s_* of the sensitive element can be expressed as:

(2) $$R_{s}=\rho L_{2}/w(d_{0}-x)$$

where *L*_2_ is the length of the sensitive element, *x* is the corrosion depth of the element. When no corrosion happens, the ratio of the *R_c_*_0_ and *R_s_*_0_ can be described as:

(3) $$k_{0}=R_{c0}/R_{s0}=L_{1}/L_{2}$$

Through the measurement ratio of *R_c_* and *R_s_*, the corrosion depth can be calculated:

(4) $$x=d_{0}(1-k_{t}/k_{0})$$

where *k_t_* is the measurement resistance ratio of *R_c_* and *R_s_*. Therefore, it can be found that the difference between the *L*_1_ and *L*_2_ makes no influence on the corrosion depth measurement result. The corrosion rate of each coupon can be reflected by the slopes of the corrosion depth curves.

During the corrosion depths measurements, Switch 1 and Switch 2 were kept open. The ER of the elements on the three electronic coupons can be measured in sequence through the autoswitch system of RM 3545. CS EC 1 was only used for corrosion depth measurement. For the galvanic current and coupled voltage measurements, the autoswitch system of RM 3545 was kept open and Switch 1 and Switch 2 were switched on. The CS EC 2 and SS EC 3 were coupled during this period. The galvanic current between the two coupons and the coupled potential of the coupons can be measured. The monitoring frequency was 1 Hz in the test. The cycle periods for corrosion depth measurements were 2 h and the measurement duration was less than 10 s. For the other time, galvanic current and coupled potential signals were kept for continuous monitoring. Since the variation of the ER signal is only a correlation to the change of the cross-section area [33], the corrosion depths of the electronic coupons can be actually reflected without considering other interferences such as the conductivity of the concrete medium or the reaction of the rust layer on the steel surface. In the long-term rebar corrosion monitoring test, due to the short duration of the ER measurements, the galvanic current can seem to be continuously monitoring without interruption. Thus, the corrosion depths and the galvanic current can be thought approximately to be measured synchronously by the new design galvanic sensor system.

### 2.3. Electrochemical Measurements

**Figure 3:**
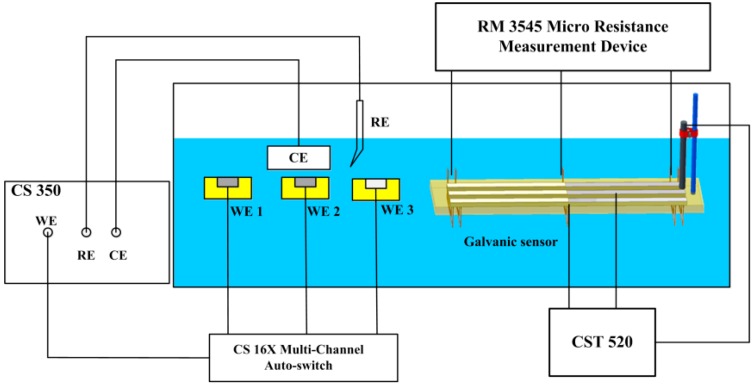
Test cell of the corrosion monitoring system.

The corrosion monitoring system in the test cell is shown in Figure 3. Besides the galvanic sensor system, a traditional three-electrochemical cell was used for the LPR and polarization curves measurements. Three working electrodes (WEs) were used in the tests. WE 1 and WE 2 were CS electrodes and WE 3 was an SS electrode. WE 1 was used for the LPR measurement and WE 2 and WE 3 were used for polarization curves measurements. A platinum plate was used as a counter electrode and a saturated calomel electrode was used as the reference electrode. LPR measurements were performed by applying anodic voltage scans at the rate of 0.1 mV/s over a range of ±10 mV around the stabilized open-circuit potential (OCP). Polarization curves were measured by scanning the potential, approximately from −500 mV to 750 mV (vs. SCE). The three working electrodes were alternately connected to the electrochemical station CS 350 (CorroTest, Wuhan, China) for the LPR and polarization curves measurements through the CS 16X (CorroTest, Wuhan, China) multi-channel auto switches.

### 2.4. Experimental Procedures

**Figure 4:**
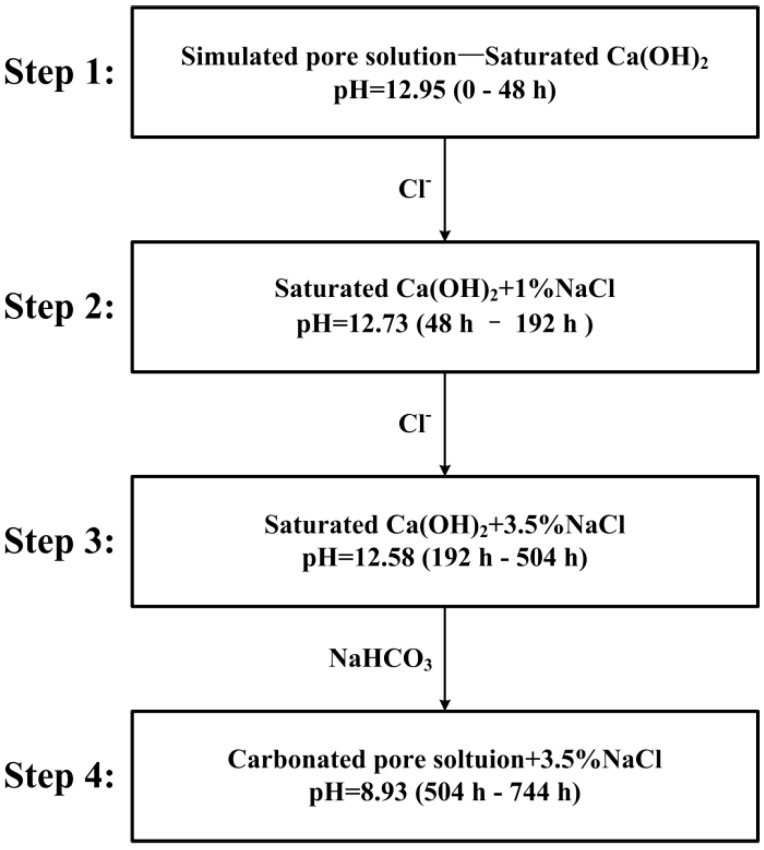
Scheme of the experimental procedures.

The test cell was placed in an air-conditioned room with temperature fluctuation at 19 ± 1.5 °C. The test solution was directly exposed to the air and the pH of the solution was monitored by a TDS YM 2712 pH monitor. The saturated Ca(OH)_2_ aqueous solution with pH = 12.95 was firstly used as a simulated pore solution and then successively modified by the addition of chloride ions (1% and 3.5% NaCl solution). Following, the pH of the simulated pore solution was adjusted to 8.93 by the introduction of sodium bicarbonate solution to simulate the carbonated pore solution. Figure 4 presents the scheme of the four experimental procedures:

During the test, the pH had a little decrease with the introduction of the chloride ions. The whole test period was nearly 31 days and during each test procedure, new polished corrosion coupons were immersed into the test solution for surface observation. At the end of each step, the corrosion coupons were taken out and the surfaces of the corrosion coupons were observed by FEI Quanta 200 scanning electron microscope (SEM) (Hillsboro, OR, USA) and KEYENCE VH-8000 optical microscope (Osaka, Japan). After the whole test, the surface of the CS electrode (WE 1) used for the LPR measurement was also observed by SEM. The morphologies of the electronic coupons were observed by KEYENCE VH-8000 microscope.

## 3. Experimental Results

### 3.1. Measurement Results of the Galvanic Sensor System

**Figure 5:**
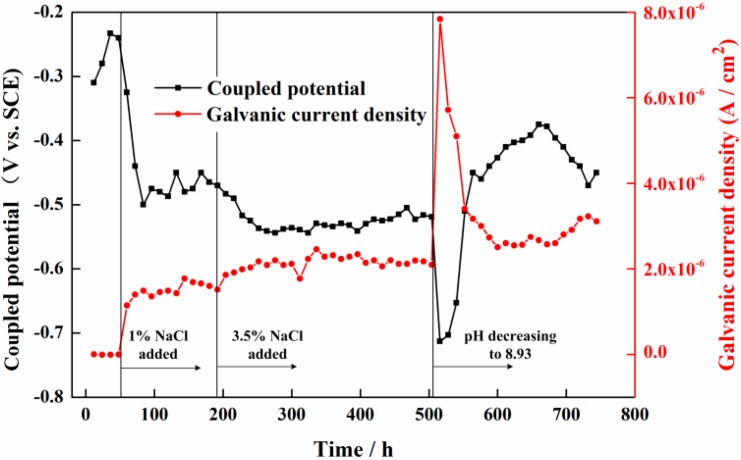
Time dependence of the coupled potential and the galvanic current density variations between the CS EC 2 and SS EC 3.

The coupled potential and galvanic current density variations between the CS EC 2 and SS EC 3 are shown in Figure 5. It is seen that the coupled potential reached −240 mV_SCE_ after the sensor was immersed in pure Ca(OH)_2_ solution for 48 h. The galvanic current was negative, which means the CS EC 2 had a higher potential than the SS EC 3 and acted as a cathode. The galvanic current density was close to −1.17 × 10^−8^ A/cm^2^, which nearly had no influence on the corrosion processes. After 1% NaCl was added to the solution, the coupled potential had a significant decrease from −240 mV_SCE_ to −480 mV_SCE_ in 36 h and came to a stable value of −470 mV_SCE_. The galvanic current also changed to positive and had a large increase to 1.6 × 10^−6^ A/cm^2^. It indicates that the breakdown of the passive film on the CS EC 2 and it turned into an anode after the introduction of chloride ions. The coupled potential of the sensor had a further decrease to −530 mV_SCE_ after the concentration of NaCl increased to 3.5%. The galvanic current density also had a slight increase to 2.3 × 10^−6^ A/cm^2^. When the pH of the solution decreased to 8.93, the coupled potential had a sudden reduction to −713 mV_SCE_ in 12 h and the galvanic current density also had a dramatic increase to 7.8 × 10^−6^ A/cm^2^. Following, the coupled potential gradually recovered and became stable at −400 mV_SCE_, which was even higher than that in high pH conditions with NaCl added. The galvanic current density also decreased to a stable value of 3.13 × 10^−6^ A/cm^2^, which is higher than that in the previous condition.

**Figure 6:**
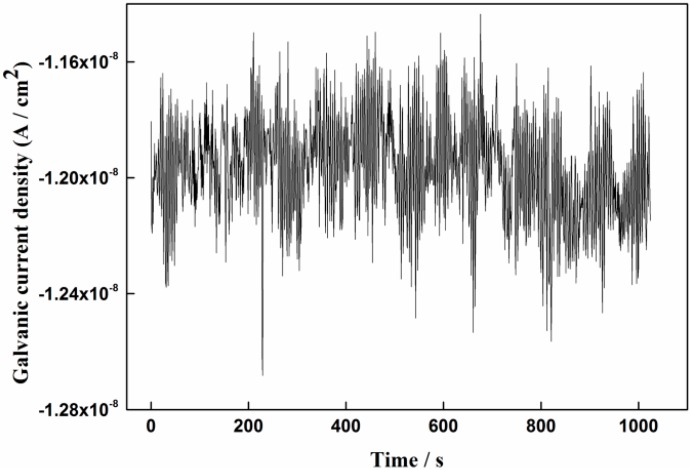
Galvanic current density monitored by the sensor after immersed in pure Ca(OH)_2_ solution for 36 h.

**Figure 7:**
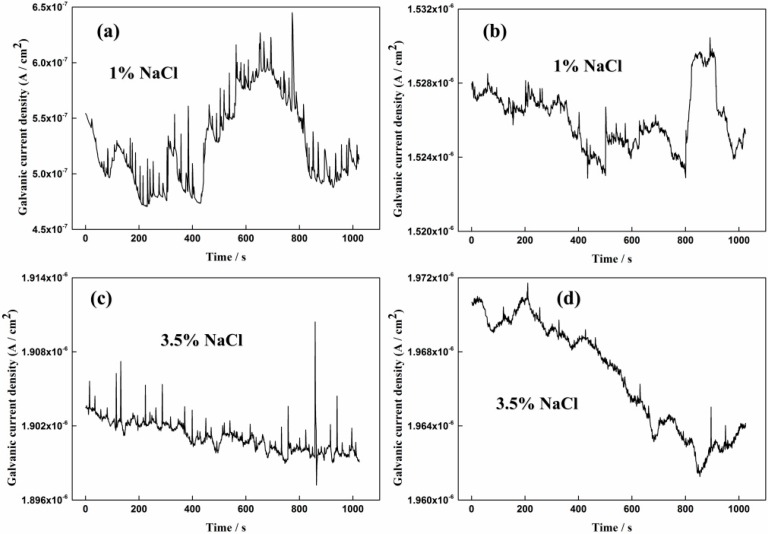
The galvanic current density in different concentrations of solutions containing chloride ions (**a**) after 1% NaCl added for 5 h (**b**) after 1% NaCl added for 120 h (**c**) after 3.5% NaCl added for 15 h (**d**) after 3.5% NaCl added for 252 h.

**Figure 8:**
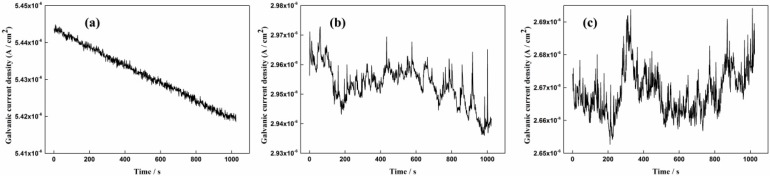
The galvanic current density monitored by the sensor with pH decreased to 8.93; (**a**) after pH decreased for 24 h; (**b**) after pH decreased for 82 h; (**c**) after pH decreased for 180 h.

More detailed information of the galvanic current noise during different test periods is shown from Figure 6 to Figure 8. It can be seen from Figure 6 that the current fluctuations were small and no current transients are found, which means the passive film is integrated. As shown in Figure 7a, the current transients can be clearly observed after 1% NaCl was added for 5 h. The fluctuations of the current spikes reached nearly 4 × 10^−8^ A/cm^2^. It is seen from Figure 7b that the galvanic current density increased to 1.53 × 10^−6^ A/cm^2^ after 1% NaCl was added for 120 h. However, the current fluctuation had a slight decrease, which might indicate the forming of a rust layer or stable pits on the CS EC 2 [34]. The same phenomenon can be seen from Figure 7c,d, that the fluctuation of the current became intensive and frequent after the concentration of NaCl increased to 3.5% at the initial 15 h and that the current fluctuations became smaller after 3.5% NaCl was added for 252 h.

It is seen from Figure 8a that current transients disappeared after pH was decreased to 8.93 for 24 h, which indicates that pitting corrosion might change into general corrosion on the CS EC 2 during this period. However, with the decrease of the galvanic current density and the rise of the coupled potential after pH was decreased for 82 h, current spikes are observed again from Figure 8b, which means localized corrosion re-emerging on the CS EC 2. It can be seen from Figure 8c, after the pH was decreased for 180 h, the frequency of the current spikes had a decrease and the fluctuations of the galvanic current had nearly no changes compared with that shown in Figure 8b.

**Figure 9:**
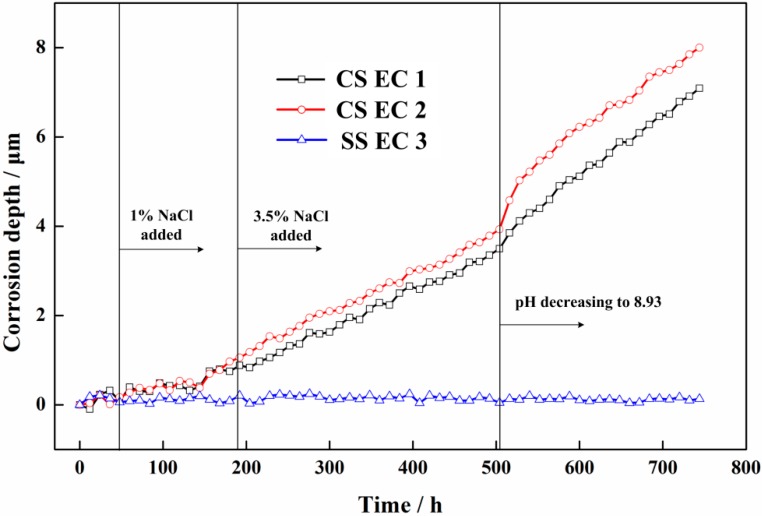
The corrosion depths of the three electronic coupons measured by the ER technique.

Figure 9 shows the corrosion depths of the three electronic coupons measured by the sensor system. It is seen that no corrosion happened on the SS EC 3 through the whole test. The corrosion rates of the CS EC 1 and CS EC 2 were close to zero in pure Ca(OH)_2_ solution, and the initial period in 1% NaCl contained pore solution. After 1% NaCl was added for 156 h, obvious corrosion depth increase of the CS EC 2 can be seen. The corrosion rate of the CS EC 2 reached 0.022 mm/a. Though the corrosion depth of the CS EC 1 also had a slight increase with 1% NaCl added, the corrosion process was not stable during this period. Significant corrosion processes can be found after the concentration of the NaCl increased from 1% to 3.5%. The corrosion depth curves of both the CS EC 1 and 2 showed a linear growth, suggesting the corrosion rates measured by the ER signal were stable. The corrosion rates of the CS EC 1 and EC 2 were 0.077 mm/a and 0.081 mm/a, respectively. The corrosion rate of the CS EC 2 coupled with the SS EC 3 was slightly higher than that of the CS EC 1. After the pH of the solution decreased to 8.93, the corrosion rate of the CS EC 2 had a sudden increase to 0.211 mm/a in the first 24 h and gradually reduced to a stable value of 0.114 mm/a. The corrosion rate of the CS EC 1 also had an increase to the peak value of 0.162 mm/a and came to a stable value of 0.102 mm/a. It can be found from the ER measurement results that the corrosion rates of the two CS ECs only had obvious differences at the end of the 1% NaCl added period and the initial period of the decreasing of the pH to 8.93. The stable corrosion rates of both the CS ECs were very close during different test conditions, indicating the galvanic current seemed to be having no obvious impact on the corrosion process of the carbon steel.

### 3.2. Electrochemical Measurement Results

**Figure 10:**
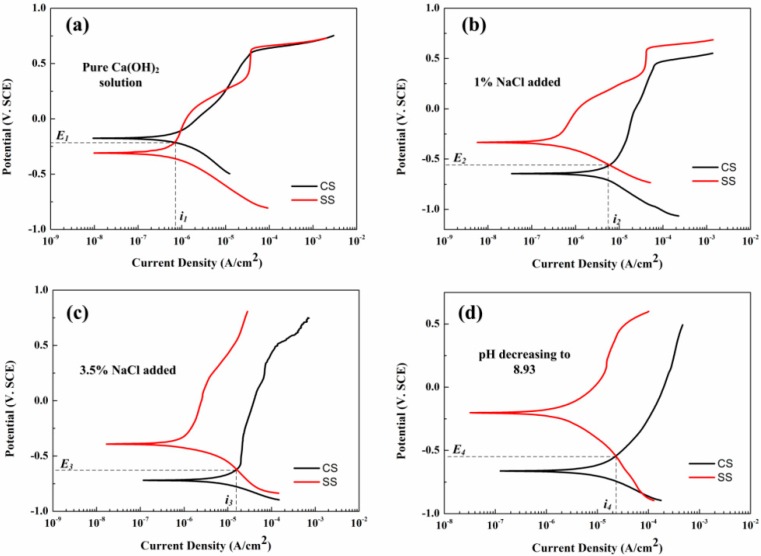
The polarization curves of the CS and SS WEs in different test conditions (**a**) pure saturated Ca(OH)_2_ solution (**b**) with 1% NaCl added (**c**) with 3.5% NaCl added (**d**) with pH decreasing to 8.93.

Figure 10 shows the polarization curves of the CS and SS WEs in different solution conditions. The corrosion potential and corrosion rates of the working electrodes fitted from the polarization curves are listed in Table 2. The corrosion potential and corrosion current were obtained by the Tafel extrapolation method. It can be seen from the polarization curves of the SS that only a slight change can be observed with 1% NaCl added to the solution. However, the corrosion potential of the CS decreased from −0.18 V_SCE_ to −0.64 V_SCE_ after 1% NaCl was added, indicating the breakdown of the passive film on the steel surface. When the concentration of NaCl rose to 3.5%, the corrosion potential of the CS and SS both had dramatic decreases to −0.71 V_SCE_ and −0.31 V_SCE_, respectively. The corrosion rates of the CS and SS also increased to 0.17 mm/a and 0.0071 mm/a. With the decreasing of the pH, the corrosion potentials of both steels recovered to −0.66 V_SCE_ and −0.26 V_SCE_, respectively. The corrosion rates of the CS working electrode nearly had no changes with the decreasing of pH. Nevertheless, the corrosion rate of the SS increased to 0.019 mm/a in this situation.

In the pure saturated Ca(OH)_2_ solution, the corrosion potential of SS was lower than CS, suggesting that CS will work as a cathode and SS will act as an anode when they are coupled. With the introduction of chloride ions, the passive film on the CS surface was broken. The corrosion potential of CS became much lower than SS showing the exchange of the anode and cathode. If the two kinds of steel are coupled in chloride-containing pore solutions, the potentials of both steels will be polarized to the same coupling potential [35]. The galvanic current and coupled potential can be estimated through the intersections of the polarization curves. The coordinates of the intersections, shown in Figure 10a–d, are listed in Table 1. It is seen from Figure 10 and Table 2, that the galvanic currents increased in turn following the changes of the solution conditions (*i_4_* > *i_3_* > *i_2_* > *i_1_*). The coupled potential firstly had a significant decrease after the introduction of chloride ions, however, the coupled potential had a slight recovery after the pH decreased to 8.93 (*E_3_* > *E_4_* > *E_2_* > *E_1_*). The variations of both galvanic current and coupled potential deduced from the intersections had the same trends as the galvanic sensor measurement results.

**Figure 11:**
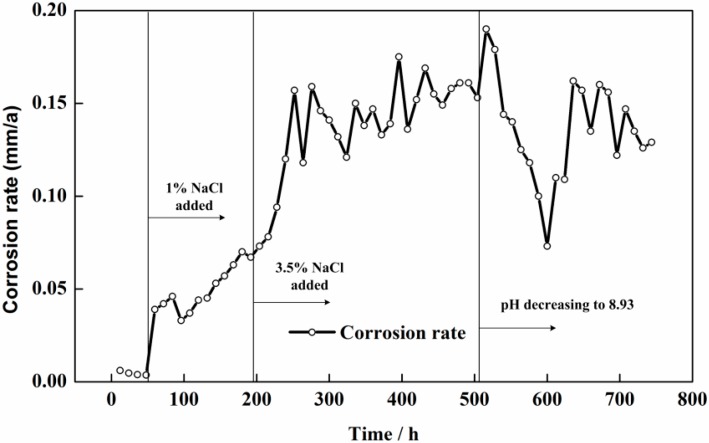
Time dependence of the corrosion rates measured by the LPR method.

Figure 11 shows the corrosion rates of CS measured by the LPR method in different solution conditions. The corrosion rate was calculated through the Stern–Geary equation [36]:

(5) $$i_{corr}=B/R_{p}$$

where *i_corr_* is the corrosion current density, B is the Stern constant (52 mV for passive state and 26 mV for active state) [37,38], and *R_p_* is calculated from the slope of the linear polarization curve. It can be seen from Figure 11 that the corrosion rate measured by LPR was close to zero in pure saturated Ca(OH)_2_ solution. The corrosion rate of the CS electrode had a sudden increase to 0.042 mm/a and gradually increased to 0.067 mm/a. The LPR measured results was nearly 3 times higher than that measured by the CS EC 2. A dramatic increase of the corrosion rate from 0.067 mm/a to 0.15 mm/a can be seen, with the concentration of NaCl increased to 3.5% for 60 h. The measurement result was still 2 times higher than the data measured by the corrosion coupons. The LPR measurement result had a large fluctuation, with the pH of the solution decreased to 8.93. The corrosion rate firstly reached the highest value of 0.19 mm/a and soon decreased to 0.08 mm/a. The corrosion rate measured by the LPR method was close to the results measured by electronic coupons during this period. Following, the corrosion rate measured by the LPR method recovered to a stable value of 0.13 mm/a at the end of the test, which was still higher than the ER measurement results.

### 3.3. Surface Characterization

**Figure 12:**
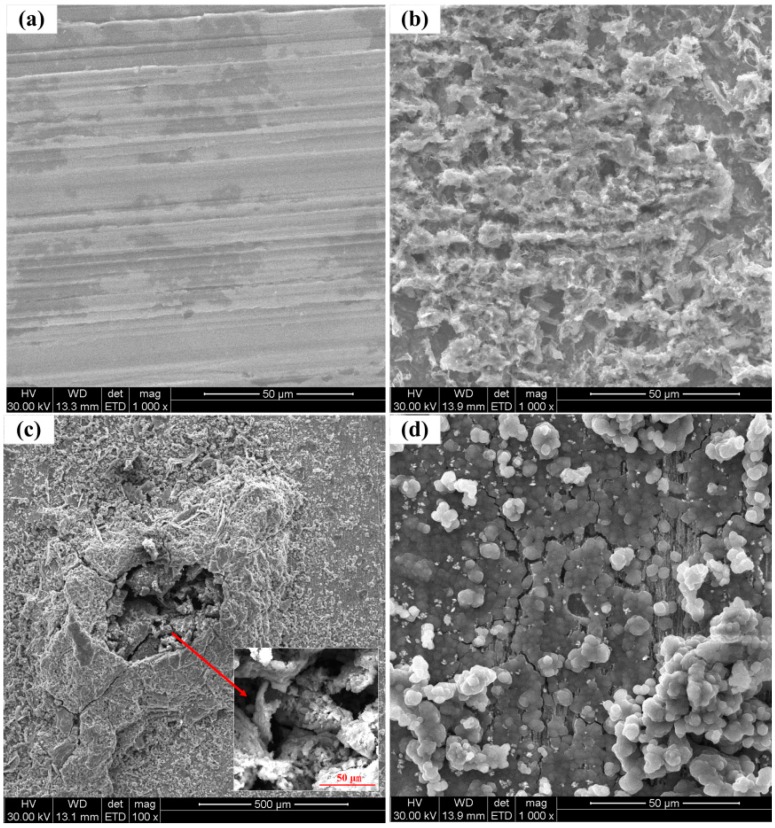
SEM images of the corrosion coupons’ surfaces after corrosion in different solution conditions, (**a**) pure saturated Ca(OH)_2_ solution (**b**) saturated Ca(OH)_2_ solution with 1% NaCl (**c**) saturated Ca(OH)_2_ solution with 3.5% NaCl (**d**) with pH decreasing to 8.93.

Figure 12 shows the SEM views of the surface morphologies of the corrosion coupons. The corrosion coupons were independently immersed in different solution conditions and carried out before the changes of the solution conditions. As seen from Figure 12a, the surface of the corrosion coupon is smooth, and the scratches can be clearly seen, indicating the steel was well-protected in the saturated Ca(OH)_2_. As shown in Figure 12b, a porous rust layer was formed on the steel surface, which means the breakdown of the passive film and obvious corrosion happened after 1% NaCl was added. The porous rust layer can also be seen from Figure 12c, and serious localized corrosion is found on the steel surface. It suggests that the steel will suffer stable pitting corrosion in 3.5% NaCl-containing pore solution. As shown in Figure 12d, a more dense corrosion product film can be seen on the steel surface with the pH of the solution decreased to 8.93.

**Figure 13:**
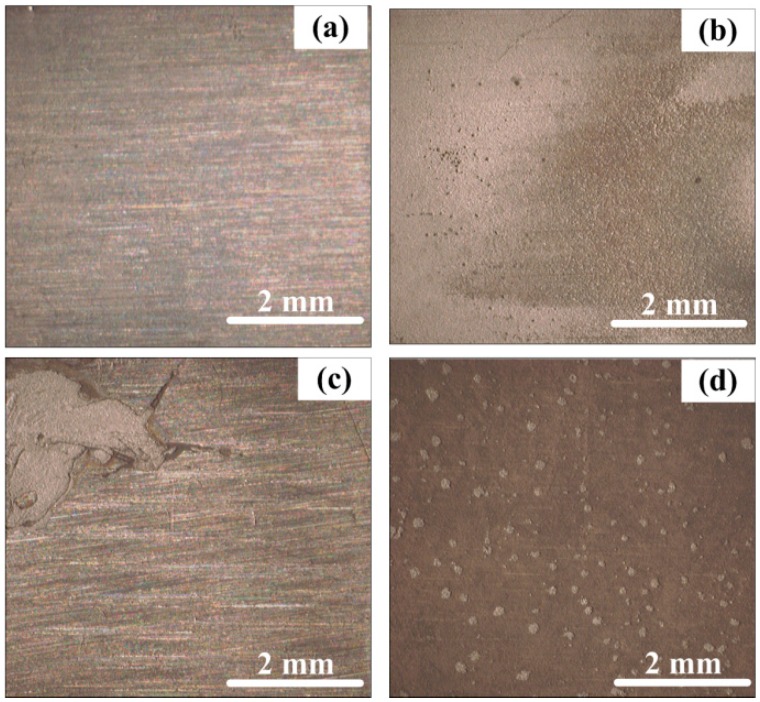
The surface morphologies of the corrosion coupons in different solution conditions after the rust layers were removed by rubber (**a**) pure saturated Ca(OH)_2_ solution (**b**) saturated Ca(OH)_2_ solution with 1% NaCl (**c**) saturated Ca(OH)_2_ solution with 3.5% NaCl (**d**) with pH decreasing to 8.93.

Figure 13 shows the surface morphologies of the corrosion coupons after the porous rust layers were removed by rubber. Tiny pits can be seen from Figure 13b, indicating a metastable pitting corrosion process happened on the steel surface in 1% NaCl-containing pore solution. However, the whole surface of the steel also became rough, suggesting general corrosion also occurred on the steel surface. Obvious localized corrosion behavior can be found from Figure 13c. It demonstrates that stable localized corrosion is liable to form in a 3.5% NaCl solution condition. It is seen from Figure 13d that the rust layer cannot be completely removed by rubber, which means the corrosion product layer had a well-adsorptive performance. However, tiny pits can also be found on the steel surface, suggesting that the metastable pitting corrosion also occurred along with the general corrosion when the pH decreased to 8.93.

**Figure 14:**
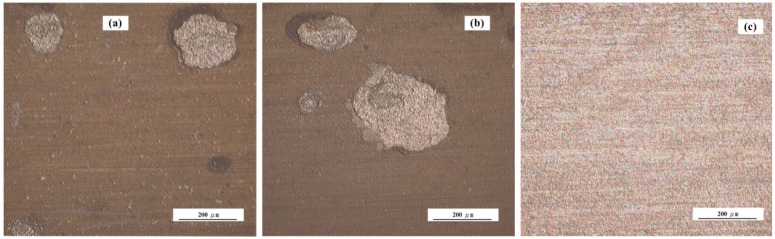
Surface morphologies of the electronic coupons (**a**) CS EC 1 (**b**) CS EC 2 (**c**) SS EC 3.

**Figure 15:**
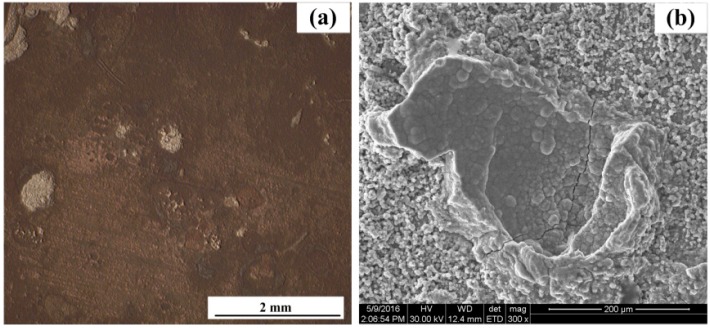
The surface morphology and SEM image of the WE used for the LPR measurement (**a**) the surface observed by optical microscope (**b**) SEM image of pitting corrosion area.

Figure 14 shows the surface morphologies of the three electronic coupons after corrosion for 744 h. The porous rust layers on the CS EC 1 and CS EC 2 were also removed. Significant localized corrosion patterns can be found on both CS WEs. Larger pits can be found on the CS EC 2, indicating the localized corrosion on the CS EC 2 was a more serious than that on the CS EC 1. The surface of the SS EC 3 was smooth and flat, suggesting the low corrosion rate of the stainless steel. Figure 15 shows the surface morphology of the electrode used for the LPR test. The surface of the electrode was rough and serious pitting corrosion can be seen from the SEM image, as shown in Figure 15b. It reveals that slight polarization may lead to the acceleration of localized corrosion in chloride-containing pore solution.

## 4. Discussions

### 4.1. The Corrosion Processes in Different Solution Conditions

The corrosion processes of the rebar in different pore solution conditions were quite different. As shown in Figure 7a, the frequent current transients reveal a metastable pitting corrosion process in 1% NaCl-containing pore solution. The tiny pits shown in Figure 13b verify the corrosion process. When the concentration of the NaCl reaches 3.5%, metastable pitting corrosion will become active again at the initial period, which can be seen from Figure 7c. The stable localized corrosion will soon form on the steel surface and it can also lead to the decrease of the current transients [34]. The serious localized corrosion on the surfaces of the corrosion coupon and the CS EC 2 also shows the stable pitting corrosion process during this period. When the pH of the solution decreases to 8.93, the disappearance of the current spikes reflects the corrosion patterns changing into general corrosion. The corrosion rates measured by both the ER and LPR methods have significant increases at the initial period with pH decreased to 8.93. The passive film on the CS EC 2 cannot be maintained in the low pH solution. Due to the overall destruction of the passive film, the galvanic current had an intensive increase. A new corrosion product layer will soon form on the steel face due to the high corrosion rate. The new corrosion product layer is more dense than that in high pH solution conditions and it will perform a mitigation effect on the corrosion rate. However, the crevice and default on the layer shown in Figure 12d indicate the inhomogeneity of the new corrosion product film. It leads to the metastable pitting corrosion happening again, which can be deduced from the reappearance of the current transients. The small pits shown in Figure 13d have a good correlation with the galvanic current noise changes. The test results show that the corrosion processes can be well-revealed by the galvanic sensor.

### 4.2. The Relationship between the Galvanic Current and the Corrosion Current Measured by the LPR Method

**Figure 16:**
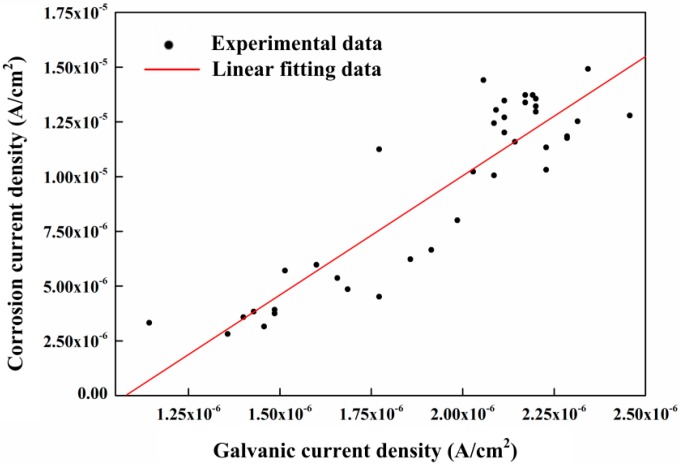
The relationship between the corrosion current density measured by LPR and the galvanic current density between CS and SS in chloride-containing saturated Ca(OH)_2_ solution conditions.

The previous studies revealed that the galvanic current between CS and SS had a strong linear correlation to the corrosion current measured by the LPR method [28]. Through the comparation of the galvanic current shown in Figure 5 and the corrosion rate shown in Figure 11, it can be found that the galvanic current in pure saturated pore solution is negative and the corrosion rate is close to zero. They have no obvious correlation in this solution condition. It is seen from Figure 5 that the galvanic current density comes to a stable value of 3 × 10^−6^ A/cm^2^ at the end of test in a low pH solution condition. However, the corrosion rate measured by the LPR method has a large fluctuation from 0.13 mm/a to 0.16 mm/a, indicating there is also no obvious correlation during this period. The corrosion rate and the galvanic current only have similar variation trends with the introduction of chloride ions in saturated Ca(OH)_2_ solution. The relationship of the corrosion current density and the galvanic current density during this period is shown in Figure 16. The corrosion current density and the galvanic current density can be expressed as:

(6) $$i_{corr}=10.86i_{g}-10^{-5}$$

where *i_corr_* is the corrosion current density and *i_g_* is the galvanic current density. The *R^2^* (*R* is the correlation coefficient) of the linear fitting data is 0.84, indicating an excellent linear correlation between the galvanic current and corrosion current. It shows the corrosion current can be calculated from the galvanic current in chloride-containing saturated Ca(OH)_2_ solution. The corrosion rate also can be represented by the galvanic current in this condition. However, it is not suitable for the carbonated concrete conditions.

### 4.3. The Corrosion Rates Measurement Differences between the Electronic Coupons and the LPR Method

It can be obtained from the test results that the corrosion rate measured by the ER method is quite different from that measured by the LPR method. In 1% NaCl-containing pore solution, the electronic coupons only have a weak response to the corrosion depth changes. As the corrosion behavior is metastable pitting corrosion during this period, the tiny pits forming on the steel surface have nearly no influence on the change of the cross-section area. Therefore, the corrosion rate measured by the ER method is close to zero. When the concentration of NaCl increases to 3.5%, stable localized corrosion occurs on the steel surface. The stable localized corrosion will lead to an obvious change of the cross section area and it can be clearly revealed by the electronic coupons. However, since the local cross-section area change is converted to the change of the whole steel surface during corrosion depth calculation, the corrosion rate measured by the ER method is still lower than the real localized corrosion rate. During this period, the corrosion rate measured by LPR was almost 2~3 times higher than that measured by the electronic coupons. However, something interesting can be found after the pH of the solution decreased to 8.93. Through the previous analysis of the galvanic current noise, the corrosion behavior changed into general corrosion at the beginning of the period after NaHCO_3_ was added to the solution. The corrosion rate measured by CS EC 1 and CS EC 2 firstly reached the peak values of 0.162 mm/a and 0.211 mm/a, and gradually decreased to stable values of 0.102 mm/a and 0.114 mm/a. The corrosion rate measured by the LPR method firstly reached the highest value of 0.19 mm/a and soon decreased to 0.09 mm/a, which continued for 48 h. It can be found that the corrosion rates and the variation trends measured by both methods are close when general corrosion occurs on the steel surface. However, when metastable pitting corrosion or localized corrosion happen on the steel surface, a much higher corrosion rate will be measured by the LPR method. It can be found that, with the reappearance of the metastable pitting corrosion on the steel surface, the LPR measurement result increases to 0.16 mm/a. The corrosion rate has a large fluctuation due to the unstable corrosion behavior on the steel surface, which can be deduced from the noise signal shown in Figure 8c,d.

Through the observation of the morphologies, more serious pitting corrosion can be seen on the electrode used for the LPR test. It indicates that the WE used for the LPR test will suffer more serious localized corrosion with an anodic polarization process. As it is well known that, with the introduction of the chloride ions into the solution, the FeOOH passive layer will be reduced into Fe_3_O_4_ or Fe_2_O_3_, which is less protective. The reduction of the FeOOH will cause the local breakdown of the passive film and lead to the initial pitting corrosion process [24]. Once a pit is initiated on the steel surface, the aggressive ions will soon come into the occluded site and cause continuous localized corrosion due to the self-sustaining effect [39]. The occluded pit area will have a more negative potential than the other areas on the steel surface. However, during the LPR measurement period, the applied potential is based on the open circuit potential of the whole electrode. Therefore, the pitting area may suffer more serious anodic polarization, and performs high anodic current in this situation. It will result in the corrosion current measured by the LPR method being higher than the real corrosion current. Consequently, the real corrosion rate of the steel is between the ER and LPR measurement results.

### 4.4. The Galvanic Corrosion Behavior between CS and SS

**Figure 17:**
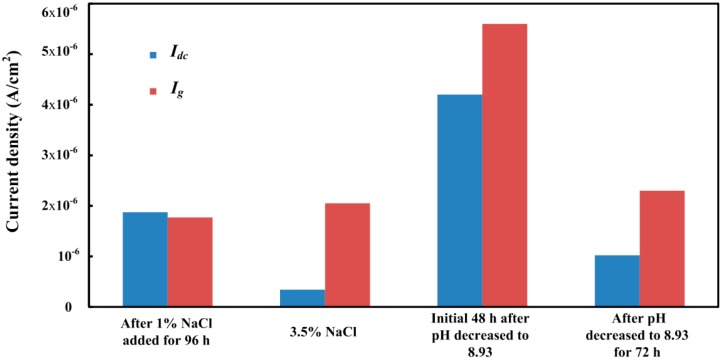
The comparison diagram of the calculated corrosion current density difference and the galvanic current density in different solution conditions.

The acceleration effect of the galvanic corrosion between CS and SS can be further studied by the relationship between the galvanic current density and the corrosion rate difference between the CS EC 1 and CS EC 2. The difference of the corrosion current density (*I_dc_*) can be calculated from the corrosion rates difference [40] between the CS EC 1 and CS EC 2:

(7) $$I_{dc}=nF\rho \Delta v/M$$

where Δ*v* is corrosion rate difference between the CS EC 1 and CS EC 2, *n* is valence, F is Faraday constant and *ρ* is the density of the metal. It is noted that the corrosion rate difference is measured by the ER method. Thus, the corrosion current difference is only used to represent the corrosion current difference on general corrosion behavior. Due to the resolution of the ER method, the corrosion rate cannot be monitored in pure saturated Ca(OH)_2_ solution. After 1% NaCl was added for 96 h, the *I_dc_* was nearly the same as *I_g_*, suggesting that the galvanic current also contributes to the general corrosion on the CS EC 2. However, because the corrosion rate measured by the CS EC 1 is not stable during this period, the comparison between *I_dc_* and *I_g_* may not be accurate. As shown in Figure 17, it can be clearly seen in 3.5% NaCl-containing pore solution that only less than one fifth galvanic current contributed to the general corrosion rate. It is known that the CS is suffering localized corrosion during this period, which indicates that the galvanic current is not provided by the whole surface of the CS EC 2. Most of the galvanic current should be provided by the pitting corrosion area. When the corrosion process transferred to uniform corrosion after the pH of the solution decreased to 8.93, it can be seen that *I_dc_* occupied three quarters of *I_g_*, suggesting that most of the galvanic current will contribute to the general corrosion. Following, with localized corrosion reappearing on the steel surface, the proportion of *I_dc_* decreased to 44 percent. The test results reveal that, when localized corrosion happens on the steel surface, most of the galvanic current will come from the pitting corrosion area. In other words, the galvanic corrosion behavior between CS and SS may lead to the acceleration of localized corrosion in some conditions. It also well explains why more serious pitting corrosion appeared on the surface of the CS EC 2. The monitoring data provided by the new design galvanic sensor shows a new analytical method for the evaluation of localized corrosion.

## 5. Conclusions

The investigation of the new design CS/SS galvanic sensor for the monitoring of rebar corrosion in different pore solution conditions shows the following results:

1. Through the special designs of the electronic coupons and the measurement circuit, the ER method can be used in combination with the ZRA technique. The galvanic current between the CS coupon and the SS coupon and the corrosion depths of the electronic coupons can be monitored simultaneously. More information on the rebar corrosion can be obtained by the sensor system.
2. The galvanic current noise can directly reflect the corrosion processes of the rebar in different solution conditions. The rebar trends to occur metastable pitting corrosion and localized corrosion in 1% NaCl-containing and 3.5% NaCl-containing saturated Ca(OH)_2_ solutions, respectively. The corrosion process of the steel will transfer to general corrosion at the initial period in chloride-containing carbonated pore solution. With the forming of the new dense product layer on the steel surface, localized corrosion will appear on the steel surface again.
3. The ER method provides excellent responses to general corrosion process. However, it has no response to the metastable corrosion process, and the corrosion rate measured by the ER method is lower than the real value during stable localized corrosion processes. The LPR method will lead to the acceleration of the local anode dissolution when localized corrosion is occurring on the steel surface. Therefore, the corrosion rate measured by the LPR method is higher than the real corrosion rate in this situation. The accurate corrosion rate is between the measurement results obtained by the ER and LPR methods when localized corrosion happens.
4. The galvanic current density between CS and SS shows a strong linear correlation to the corrosion current density measured by the LPR method in chloride-containing saturated Ca(OH)_2_ solution. It indicates the galvanic current can be used as an index for the evaluation of the corrosion rate when chloride ions exist in the pore solution. However, this relationship is not suitable for the carbonated pore solution.
5. Through the comparison of the corrosion current difference between CS electronic coupons and the galvanic current, the corrosion behavior can be qualitatively assessed. When the corrosion current difference occupies most part of the galvanic current, the corrosion process tends to be a general corrosion process. However, if the galvanic current is much higher than the corrosion current difference, it indicates a serious localized corrosion process is occurring on the steel surface. It also can be used as an indication for the evaluation of localized corrosion in concrete.

## Figures and Tables

**Table 1:** Chemical compositions (weight percent) of the Q235 carbon steel and 316L stainless steel.

	C	Si	Mn	P	S	Mo	Cr	Ni	Fe
Q235	0.22%	0.30%	0.40%	0.045%	0.04%	/	0.01%	0.01%	Bal.
316L	0.03%	1.00%	2.00%	0.035%	0.03%	2.50%	17.00%	12.60%	Bal.

**Table 2:** Results of corrosion potential and corrosion rate measurements and the intersections of the CS and SS polarization curves in different solution conditions.

Solution Conditions	Steel Kinds	*E_corr_*/V_SCE_	Corrosion Rate/(mm/a)	Intersections of CS and SS	
				Potential (V_SCE_)	Current Density (A/cm^2^)
Pure Ca(OH)_2_	CS	−0.18	0.0091	−0.21	4.3 × 10^−^^7^
	SS	−0.27	0.0022		
With 1% NaCl added	CS	−0.64	0.14	−0.51	6.3 × 10^−^^6^
	SS	−0.31	0.0041		
With 3.5% NaCl added	CS	−0.71	0.17	−0.63	1.7 × 10^−^^5^
	SS	−0.37	0.0071		
With pH decreasing to 8.93	CS	−0.66	0.18	−0.57	2.2 × 10^−^^5^
	SS	−0.26	0.019		
